# The effect of health professionals’ perceptions of organizational impediments on emotional labor and job satisfaction

**DOI:** 10.3389/fpsyg.2025.1537830

**Published:** 2025-03-27

**Authors:** Hülya Gündüz Çekmecelioğlu, Jale Balkaş, Sabiha Sevinç Altaş, Döndü Sevimli Güler

**Affiliations:** ^1^Department of Business Administration, Kocaeli University, İzmit, Türkiye; ^2^Vocational School of Health Services, Sakarya University, Sakarya, Türkiye; ^3^Department of Nursing, Sakarya University of Applied Sciences, Sakarya, Türkiye

**Keywords:** emotional labor, organizational impediments, job satisfaction, JD-R theory, COR theory

## Abstract

**Introduction:**

Healthcare professionals are often required to manage their emotions effectively within the workplace, a task that can contribute to considerable stress and psychological strain. Emotional displays, which can be affected by organizational impediments, can affect healthcare workers’ levels of job satisfaction.

**Materials and methods:**

The study adopts a cross-sectional research design. Data were collected from 651 healthcare professionals in Turkey through a convenience sampling method, utilizing online and face-to-face survey questionnaires, and analyzed using SPSS Process Macro v4.2 and AMOS v24.

**Results:**

The findings reveal that organizational impediments positively impact the surface acting and deep acting, as they compel healthcare workers to invest more effort, planning, and control in managing their emotions. However, these impediments also negatively affect job satisfaction. Furthermore, surface acting and deep acting were found to have distinct effects on job satisfaction, with surface acting serving as a mediator between organizational impediments and reduced job satisfaction.

**Conclusion:**

The findings highlight the significant role of surface behaviors in mediating the negative relationship between organizational impediments and job satisfaction, underscoring their critical impact on employee satisfaction. Moreover, the pronounced influence of deep acting on job satisfaction emphasizes the need for strategies aimed at preserving or enhancing employees’ intrinsic motivation. These insights suggest that health institutions should prioritize the development of policies designed to alleviate the emotional labor demands placed on employees. Furthermore, addressing and mitigating organizational impediments appears to be a key strategy for fostering higher levels of job satisfaction among healthcare professionals. These results were compared with findings from previous studies and discussed within the theoretical frameworks of Job Demands-Resources (JD-R) and Conservation of Resources (COR) theories.

## 1 Introduction

Rising competition in the service sector has prompted businesses to enhance the quality of services offered to customers. Service quality is commonly described as the customer’s perception of the relative excellence or inadequacy of a service provider and its offerings, often regarded as closely aligned with the customer’s overall attitude toward the organization (Prakash and Mohanty, 2013). The closer customers’ expectations align with their perceptions of service quality, the higher the perceived quality will be. A key component of perceived service quality is the communication employees have with customers (Bowen and Schneider, 1988; Schneider and Bowen, 1995). For this reason, managers across various service industries, including healthcare, prioritize how their employees interact with customers (Morris and Feldman, 1996). The effectiveness of the communication process is closely related to employees’ emotions and the nature of the work environment that affects these emotions.

Healthcare professionals operate in demanding environments. Despite their crucial role in public health, many face significant challenges worldwide. High patient volumes, grueling work schedules, and insufficient protective equipment are among healthcare professionals’ primary challenges globally (Shahbaz et al., 2021). They also contend with issues like time pressure, low wages, limited opportunities for professional development, shortages of supplies and medications, job insecurity, and inadequate compensation (Karadzinska-Bislimovska et al., 2014). Workplace violence is another serious concern for healthcare professionals. The World Health Organization (WHO) reports that 8.38% of healthcare workers experience workplace violence at least once in their careers (Zhong et al., 2022). Additionally, adapting to evolving healthcare services amid the digital revolution of the 21st century (Etheredge and Fabian, 2022) presents another layer of difficulty for healthcare professionals.

As in most countries, the working conditions for healthcare professionals in Turkey are notably challenging. With only 2.2 doctors and 2.8 nurses per 1,000 people, Turkey lags behind the OECD average of 3.7 doctors and 9.8 nurses per 1,000 people (Euronews, 2024). Due to the lack of adequate opportunities for healthcare professionals in the country, a significant number of healthcare workers, particularly doctors, are opting to migrate to nations that provide more favorable conditions (Euronews, 2024). Healthcare professionals in Turkey endure immense pressure due to the high patient load and low wages (Kocaman et al., 2018). This situation diminishes their commitment to work, weakens job performance, and hampers organizational efficiency (Bakker and Demerouti, 2008), while also degrading the quality of patient care and healthcare services overall (Karadzinska-Bislimovska et al., 2014; Zaghini et al., 2020). Improving the work environment and reducing organizational impediments for healthcare professionals can help them manage their emotions more effectively, allowing for more authentic and sincere behavior, which in turn can enhance job satisfaction. Research supports that decreasing job stress among healthcare workers improves the quality of services they provide (Karadzinska-Bislimovska et al., 2014).

This study seeks to address several critical gaps in the existing literature. While the relationship between emotional labor and job satisfaction has been widely examined, research exploring how specific job demands, such as organizational impediments, influence emotional labor behaviors remains scarce. This is particularly evident in the healthcare sector, where the impact of organizational impediments on surface and deep acting has not been thoroughly investigated. Additionally, the mediating role of emotional labor behaviors in the relationship between organizational impediments and job satisfaction has received limited attention. Given that healthcare professionals are a workforce characterized by high emotional labor demands, there is a need for more focused research addressing their unique experiences. This study empirically contributes to organizational behavior literature by elucidating how organizational impediments shape emotional labor processes, particularly in the healthcare context. Furthermore, the study examines the interplay between organizational impediments, emotional labor, and job satisfaction through the lenses of the Job Demands-Resources (JD-R) and Conservation of Resources (COR) theories, providing a novel and insightful perspective. Specifically, it offers a theoretical contribution to the literature by highlighting how organizational impediments serve as both “job demands” and catalysts for “resource loss.” Finally, the study offers practical contributions by providing actionable recommendations for developing organizational policies aimed at mitigating the emotional labor burden and enhancing the well-being of healthcare professionals.

## 2 JD-R theory and COR theory

This study is grounded in Job Demands-Resources (JD-R) model and Conservation of Resources (COR) theory. This theory developed by Bakker and Demerouti (2008) emphasizes the importance of maintaining a balance between job resources and job demands, suggesting that when these two elements are well-balanced, employees experience greater productivity, lower absenteeism, and stronger workplace relationships (Bakker and Demerouti, 2008). Conversely, an imbalance between job demands and resources can lead to decreased focus, increased failures, and higher absenteeism among employees. According to the JD-R theory, job resources are organizational or social aspects that help employees achieve their work and organizational goals. Supportive leadership and autonomy are examples of such resources (Bakker and Demerouti, 2008; Demerouti et al., 2001). On the other hand, job demands include factors like abusive management behaviors, excessive control, and overwhelming workloads, all of which contribute to a negative organizational climate. These demands can result in psychological and physical issues for employees, such as stress and emotional exhaustion (Demerouti et al., 2001).

According to the JD-R Theory, job demands refer to the physical, mental, or emotional efforts required from employees, which can lead to stress and burnout, particularly when available resources are insufficient (Bakker and Demerouti, 2008). Within this theoretical framework, organizational impediments such as factionalism, rigid hierarchical structures, and abusive supervision can be conceptualized as significant job demands that contribute to heightened job stress and increased risk of burnout. A workplace characterized by factionalism fosters an environment of insecurity, competition, and social exclusion, compelling employees to remain constantly vigilant to the shifting political dynamics within the organization. This constant attention to internal politics imposes additional emotional and cognitive burdens on employees. In the literature, organizational politics—including factionalism—are identified as stress-inducing job demands that adversely affect employees’ well-being and diminish job satisfaction (Ferris et al., 1989). Similarly, abusive supervision exerts profound psychological and emotional pressure on employees. Persistent exposure to criticism, humiliation, and injustice creates emotional dissonance, fostering chronic stress and increasing overall job demands (Tepper, 2000). Employees under abusive supervision frequently encounter emotional conflict, as they are forced to reconcile their authentic feelings with the professional behaviors expected in the workplace.

In these contexts, emotional labor emerges as both an additional job demand and a depletable resource. Emotional labor involves the regulation and display of emotions to meet organizational expectations and is particularly prevalent in sectors characterized by hierarchical structures and high interpersonal interactions, such as the service industry (Grandey, 2000). In factionalized environments, employees must continually engage in emotional labor to navigate interpersonal dynamics, manage relationships within competing groups, and maintain a façade of professionalism despite underlying tensions. This sustained emotional regulation leads to emotional exhaustion, a critical component of burnout. Brotheridge and Lee (2003) point out that continuous demands for emotional labor progressively deplete employees’ emotional resources, ultimately contributing to burnout. The emotional strain is exacerbated in environments marked by abusive supervision, where employees must suppress negative emotions in the face of unfair treatment, further accelerating emotional exhaustion and diminishing motivation.

The dual function of emotional labor—as both a demand and a resource—plays a pivotal role in intensifying job stress and lowering job satisfaction. The persistent requirement to perform emotional labor, particularly in toxic organizational climates, drains employees’ emotional capacity, leading to burnout and disengagement (Maslach and Jackson, 1981). Furthermore, the presence of organizational impediments such as factionalism and abusive management significantly hastens this depletion process. According to Spector (1997), when job demands are excessive and resources are inadequate, employees experience lower levels of job satisfaction. Emotional labor, in its role as a depleting resource, undermines employees’ satisfaction by fostering feelings of alienation and disconnection from their work. Ultimately, the compounding effects of high job demands and the erosion of emotional resources contribute to a negative work environment, characterized by elevated stress levels and diminished employee well-being.

This study also draws upon the Conservation of Resources (COR) theory, which posits that individuals are primarily motivated by the pursuit of acquiring, accumulating, and preserving resources. This pursuit fosters motivation, whereas disruptions in resource acquisition lead to stress (Hobfoll, 1988, 1989). According to the COR theory, resources are often obtained by leveraging existing ones, and the likelihood of resource acquisition increases as the availability of resources grows (Hobfoll, 1989, 2002; Hobfoll et al., 2018). The theory further asserts that the key factor driving the acquisition of personal resources is the presence of contextual resources (Miao et al., 2023). Contextual resources, which reflect workplace conditions such as supportive leadership, financial resources, and autonomy, act as catalysts for personal resources, often referred to as psychological capital. These personal resources include attributes like self-efficacy, self-esteem, hope, resilience, and optimism, all of which contribute to enhanced job satisfaction and organizational commitment (Baskındağlı and Altındağ, 2023).

The COR theory posits that individuals are highly susceptible to resource loss and that resource depletion is a primary driver of stress and burnout (Hobfoll, 1989). Within this framework, organizational impediments such as factionalism, rigid hierarchical structures, and abusive management function as environmental and social stressors that accelerate the depletion of employees’ resources. In factional and politically charged work environments, employees must remain constantly vigilant, navigating complex interpersonal dynamics and engaging in self-protective behaviors. This ongoing need for strategic maneuvering depletes both cognitive and emotional resources, contributing to chronic stress and negatively impacting employees’ physical health over time. These effects are particularly pronounced in high-stress occupations, such as healthcare, where the additional burden of organizational dysfunction can lead to physical exhaustion (Maslach and Jackson, 1981).

One of the key ways in which factionalism erodes employee well-being is by undermining social support networks within the workplace. Trust and solidarity among colleagues are critical buffers against workplace stress, yet factional environments foster division and interpersonal conflict, ultimately weakening employees’ capacity to cope with stress (Ferris et al., 1989). In occupations where emotional labor is a fundamental requirement—such as healthcare—these organizational impediments further escalate the demand for emotional regulation. When the resources needed to sustain emotional labor are insufficient, burnout intensifies. Emotional labor, which involves the regulation of emotions to meet professional expectations, is a recognized job demand that progressively depletes employees’ emotional resilience, particularly in service-oriented professions (Grandey, 2000). Healthcare professionals, for instance, must continuously regulate their emotional responses while interacting with patients and their families. However, when additional stressors—such as factionalism and abusive management—are present, this regulatory process becomes increasingly taxing, accelerating emotional exhaustion. Hobfoll (2001) emphasizes the importance of resource replenishment in mitigating the negative consequences of resource loss. However, employees operating in dysfunctional organizational climates often struggle to access the necessary support systems to restore their depleted resources, exacerbating burnout.

The combined impact of heightened emotional labor demands and resource depletion significantly contributes to burnout and diminished job satisfaction (Grandey, 2003). As previously established, high job demands coupled with insufficient resources lead to a decline in employees’ work engagement and overall job satisfaction (Spector, 1997). Notably, emotional labor, when depleted, not only diminishes satisfaction but also fosters work alienation, further disconnecting employees from their professional roles (Yıldırım and Türker, 2018). Ultimately, when employees are subject to persistent emotional strain without adequate support mechanisms, their ability to manage work-related stress deteriorates, reinforcing the cycle of burnout, dissatisfaction, and disengagement (Brotheridge and Grandey, 2002; Mesmer-Magnus et al., 2012).

## 3 Theoretical background and hypothesis development

### 3.1 Emotional labor and organizational impediments

Emotional labor is generally understood as the process by which employees express emotions prescribed by their organization when interacting with clients or other stakeholders (Morris and Feldman, 1996). These emotional displays, particularly toward customers, reflect a psychological effort to manage and regulate emotions (Grandey, 2000).

Despite various theoretical perspectives on emotional labor, the core tenet remains consistent: Individuals regulate their emotional expressions in alignment with organizational expectations (Hochschild, 1983; Ashforth and Humphrey, 1993; Morris and Feldman, 1996). Emotional labor thus entails both the regulation of emotions and their external expression in service of organizational goals. Two primary strategies for emotional regulation are commonly identified: surface acting and deep acting. Surface acting, involving the management of outward emotional expressions without internalizing the corresponding emotions, can lead to emotional dissonance and psychological strain. In contrast, deep acting, which focuses on genuinely modifying one’s emotions to align with required displays, helps reduce emotional conflict by fostering empathy and a more authentic engagement with the task at hand (Hochschild, 1983).

The differential outcomes of surface and deep acting are widely acknowledged, with both processes capable of producing either positive or negative consequences. Surface acting, for instance, is often linked to individual stress and adverse health outcomes, whereas deep acting enhances customer service quality and contributes to job satisfaction. The contrasting effects of these emotional regulation strategies provide crucial insights into institutional training and stress management interventions (Grandey, 2000). On the other hand, Ashforth and Humphrey (1993) proposed an additional dimension of emotional labor— the expression of genuine emotion. This perspective contends that emotional labor need not always be associated with negative outcomes such as emotional dissonance and burnout. In some cases, emotional labor may naturally arise from employees’ intrinsic desire to offer superior service, driven by genuine goodwill (Diefendorff et al., 2005).

The process of emotion management, or emotional labor, is significantly influenced by the work environment in which employees operate (Ashforth and Humphrey, 1993; Morris and Feldman, 1996; Brotheridge and Grandey, 2002; Grandey, 2000). A supportive work environment promotes employee responsibility and encourages constructive problem-solving behaviors, thereby enhancing organizationally beneficial actions such as assisting colleagues and mitigating risks (George and Brief, 1992; Rhoades and Eisenberger, 2002). Organizational support, defined as the provision of appropriate working conditions and resources for employee tasks (Eisenberger et al., 1986), fosters voluntary reciprocity behaviors in line with social exchange theory. According to this framework, employees are motivated to reciprocate organizational support through increased efforts on behalf of the organization (Blau, 1964). Conversely, organizational impediments—such as excessive hierarchical structures, dysfunctional conflict, heightened competition, and workplace gossip—evoke negative emotions like anxiety, worry, fear, and restlessness (Miller, 1966).

The Conservation of Resources (COR) theory, which explains the internal dynamics of motivational stress, suggests that organizational impediments, such as abusive supervision, are associated with emotional labor (Wu and Hu, 2013). Employees who frequently interact with customers and lack job autonomy are particularly prone to burnout, whereas those with greater autonomy demonstrate more positive emotional regulation (Hochschild, 1979; Grandey, 2015). COR theory posits that for employees to be motivated, they must have access to the resources necessary for achieving their goals or fostering personal development. Resource depletion or interruption is a primary cause of stress (Hobfoll, 1988, 1989; Mansour et al., 2022; Miao et al., 2023). In line with this theory, employees with high levels of resources possess sufficient energy and resources to avoid superficial emotional displays, reducing their reliance on surface acting (Lee and Ok, 2014). Recent studies, such as that of Yu et al. (2023), highlight the impact of abusive supervision on emotional labor. Investigating the tourism and hospitality sector, they found that abusive supervision had a positive effect on surface acting, acknowledging the variability of emotions and behaviors over time (Yu et al., 2023).

The emotional labor literature has explored the impact of positive organizational climate factors such as supportive management and autonomy (Grandey, 2000; Grandey et al., 2004; Ryu et al., 2020; Beğenirbaş and Yalçın, 2012; Kaygın et al., 2018; Cinnioğlu and Salha, 2017; Altaş et al., 2023). However, to our knowledge, the effect of negative organizational climate factors, like organizational impediments, on emotional labor has not yet been investigated. Several studies (Carlson et al., 2012; Wu and Hu, 2013; Yu et al., 2023) have explored the impact of abusive supervision on emotional labor. However, the topic of organizational impediments, which is central to our study, has received limited attention in the literature.

Despite the limited research directly examining the relationship between organizational impediments and emotional labor, this connection warrants further exploration. In this study, organizational impediments are conceptualized as resource deficits or interruptions within the COR framework, and they are classified as job demands that place pressure on individuals according to the JD-R model. Consequently, employees who perceive greater organizational impediments are likely to expend more effort in managing their emotions, engaging in both surface and deep acting, which in turn necessitates greater planning and emotional regulation. Based on these insights, the following hypotheses regarding the relationship between organizational impediments and emotional labor were developed for this study.

*H1*: Organizational impediments positively affect surface acting.

*H2*: Organizational impediments positively affect deep acting.

### 3.2 Emotional labor and job satisfaction

This study explores the impact of organizational impediments on emotional labor and job satisfaction, while also examining how emotional labor influences job satisfaction. A review of the emotional labor literature reveals its frequent association with outcomes such as job satisfaction, organizational commitment, and turnover intention. However, findings on the emotional labor-job satisfaction link are mixed. Some studies report a negative impact of emotional labor on job satisfaction (Heather Bulan et al., 1997; Pugliesi, 1999; Gulsen and Ozmen, 2020; Hu et al., 2018; Nguyen and Stinglhamber, 2021; Wang et al., 2022), while others suggest a positive correlation (Kammeyer-Mueller et al., 2013; Lee et al., 2016; Özen and Yüceler, 2019; Lee and Jang, 2020; Lu et al., 2021; Amissah et al., 2022; Humphrey, 2023).

Studies have generally identified a significant positive association between deep acting and job-related attitudes, including job satisfaction and organizational commitment (Xu et al., 2020). This connection is grounded in the premise that expressing positive emotions can enhance job satisfaction by fostering the internal experience of positive affect (Schaubroeck and Jones, 2000). However, research frequently highlights the negative consequences associated with employees’ suppression of emotions and efforts to conform to prescribed emotional displays, which often result in reduced job satisfaction (Hochschild, 1983; Morris and Feldman, 1996; Grandey, 2000; Diefendorff and Gosserand, 2003). For instance, Hochschild’s (1983) study on Delta Airlines flight attendants found that emotional labor contributed to adverse outcomes, including increased job stress, burnout, absenteeism, physical symptoms such as headaches, and lower job satisfaction. Empirical evidence provided by Nguyen and Stinglhamber (2021) demonstrates that surface acting adversely impacts job satisfaction. Consequently, this study focuses on the potential effects of emotional labor, particularly surface acting, which has been shown to lead to desensitization, diminished self-confidence, and burnout in employees (Hochschild, 1983). Accordingly, the following hypotheses were formulated to explore the relationships between surface and deep acting and job satisfaction.

*H3*: Surface acting negatively affects job satisfaction.

*H4*: Deep acting positively affects job satisfaction.

### 3.3 Organizational impediments and job satisfaction

According to Conservation of Resources (COR) theory, the sustained availability of contextual resources enhances individuals’ motivation, whereas impediments that disrupt these resources induce stress (Hobfoll, 1988, 1989). Organizational impediments—characterized by factionalism, rigid hierarchies, and abusive management—represent a deficiency or negative aspect of contextual resources. These impediments can hinder employees’ ability to access personal resources and develop professionally, ultimately contributing to heightened stress.

The Job Demands-Resources (JD-R) model emphasizes the critical balance between job demands and resources, positing that the physical, social, and emotional attributes of both the job and work environment can create significant tension and pressure for employees (Bakker and Demerouti, 2008). In this context, a work environment that inhibits progress or provides negative or ambiguous cues about performance expectations diminishes employees’ ability to achieve high performance standards (Walumbwa et al., 2010). Factors such as close supervision, performance-based incentives or sanctions, strict deadlines, competition, and lack of support generate continuous pressure on employees, as their performance is constantly monitored and evaluated (Meglino, 1976). Furthermore, organizational impediments escalate destructive conflicts within the workplace, diminish teamwork, and adversely affect motivation and work quality (Yıldırım and Yavan, 2008). Research has demonstrated that abusive supervision, a detrimental aspect of the organizational climate, is negatively correlated with employee commitment to work (Lyu et al., 2016) and work engagement (Yu et al., 2023). Similarly, employee performance has been shown to decline in organizations characterized by destructive competition, heightened political struggles between departments, and rigid rule enforcement, where policies are perceived more as obligations than guidelines (Özbağ et al., 2014).

It is well-established that a negative work environment can adversely influence employee attitudes. Job satisfaction is one of the most widely researched job attitudes because it has the potential to significantly affect employee well-being and behavior, and thus the success of the organization (Brendel et al., 2023). Job satisfaction, which refers to a positive or pleasurable emotional state that develops as an individual assesses their work experiences (Locke, 1976), can be negatively affected by various organizational factors, including job characteristics, work environment, management quality, excessive workload, and conflict (Brendel et al., 2023). While studies investigating the direct impact of organizational impediments on job satisfaction are relatively limited, substantial evidence suggests that certain organizational factors, including these impediments, diminish job satisfaction (e.g., Raziq and Maulabakhsh, 2015; Singh et al., 2022). For instance, Kim and Jogaratnam (2010) demonstrated that job characteristics, stress, pressure, and participatory decision-making significantly influence job satisfaction. Based on these findings, the following hypothesis was developed to explore the relationship between job satisfaction and organizational impediments, such as poor management and rigid hierarchy.

*H5*: Organizational impediments negatively affect job satisfaction.

Employees’ emotional responses can be influenced or regulated by the organization in which they are employed. When organizations strictly dictate employees’ emotional responses, the adverse effects of emotional labor tend to intensify. Conversely, when individuals are granted some autonomy over their emotional expressions, the detrimental impacts of emotional labor are mitigated (Oral and Sevinç, 2011). Given that job satisfaction is employees’ emotional responses to their jobs and work environments (Luthans, 2011), it is likely that the process of managing emotions, especially when constrained by organizational impediments influences job satisfaction levels. Such organizational constraints can lead to increased emotional labor by depleting resources, offering limited managerial support, and fostering a conflicting work environment, all of which can significantly impact employees’ job satisfaction. Lee and Jang (2020) identified that nurses’ surface actings are positively associated with increased anxiety and frustration, whereas deep actings are positively linked to higher levels of job satisfaction.

While research directly examining the impact of organizational impediments on emotional labor is lacking, there is substantial evidence indicating that a supportive organizational environment can influence the relationship between emotional labor and job satisfaction. For instance, Hur et al. (2015) demonstrated that a supportive organizational climate moderates the relationship between emotional labor and job satisfaction. Similarly, Duke et al. (2009) found that perceived organizational support mitigates the negative effects of emotional labor, thereby enhancing both job satisfaction and job performance. Based on these results and insights, the following hypotheses are proposed to explore the role of emotional labor in the impact of organizational impediments on job satisfaction.

*H6*: Surface acting mediates the negative relationship between organizational impediments and job satisfaction.

*H7*: Deep acting mediates the positive relationship between organizational impediments and job satisfaction.

**Figure 1:**
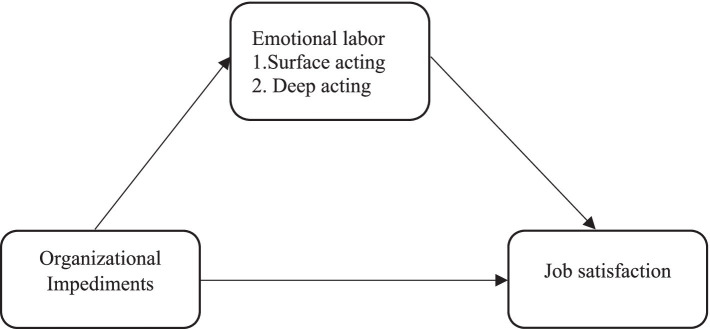
Framework of hypothesis.

The proposed hypotheses are depicted in the conceptual research model presented in Figure 1.

## 4 Materials and methods

### 4.1 Measures

To evaluate the study’s hypotheses, multi-item scales sourced from prior scholarly research were utilized to measure the different constructs being investigated. Each item was assessed using 5-point Likert scales ranging from “Strongly Disagree” (1) to “Strongly Agree” (5), reflecting respondent preferences.

In order to measure emotional labor, we used the two-dimensional (surface acting and deep acting) emotional labor scale developed by Diefendorff et al. (2005) which is composed of 11 items. Surface acting was assessed using 7 items, while deep acting was measured with 4 items from the emotional labor scale. In the original study, the scale demonstrated high internal consistency, with Cronbach’s alpha coefficients of 0.91 for surface acting and 0.82 for deep acting. In a subsequent study, Yogun (2016) conducted validity and reliability analyses of this scale within a sample of Turkish nurses. The findings indicated strong internal consistency in the Turkish context, with an overall Cronbach’s alpha of 0.87 for the emotional labor scale. These results align closely with the findings of the current study, where Cronbach’s alpha values were approximately 0.82 for surface acting and 0.81 for deep acting, reflecting a high level of reliability.

In addition, job satisfaction was measured using the short form of the Job Satisfaction Scale developed by Brayfield and Rothe (1951), as adapted by Judge et al. (2005). This version consists of 5 items and, the original study reported a Cronbach’s alpha of 0.88, indicating strong internal consistency. Although this scale has not been previously applied within the Turkish organizational context, the current study’s results demonstrated robust internal consistency, with a Cronbach’s alpha of 0.89.

Organizational impediments were measured using a four-item scale adapted from work climate scales developed by Amabile et al. (1996), Scott and Bruce (1994), and Oldham and Cummings (1996). In this study, the reliability analysis indicated a Cronbach’s alpha value of 0.74, confirming the internal consistency of the scale within this sample. For all the measures outlined above, the standard translation and back-translation procedure was applied.

Example statements for these scales are as follows:

*“I engage in a ‘show’ or ‘performance’ when interacting with patients”* (Surface acting).

*“I strive to genuinely experience the emotions required to display to patients”* (Deep acting).

*“I am reasonably content with my current job”* (Job satisfaction).

*“This institution places significant emphasis on hierarchy and adherence to established rules”* (Organizational impediments).

### 4.2 Sampling

The primary objective of this research is to reveal how organizational impediments affect the emotional labor act (surface acting and deep acting) of healthcare professionals and its reflections on their job satisfaction. The study adopts a cross-sectional research design. The data for this study were collected through questionnaire surveys utilizing the convenience sampling method. A total of 719 responses were gathered, with 245 participants completing the survey in person after receiving direct instructions, while 474 participants, informed via telephone, completed the survey through an online platform. The telephone numbers of these participants were obtained from the institutional directory. Following data screening, 68 responses were excluded due to incomplete information, resulting in a final dataset of 651 fully completed questionnaires for analysis.

To assess the adequacy of the sample size, the study employed the sample-to-item ratio criterion. According to Gorsuch (1983), a minimum ratio of 5:1 is recommended, while this study adopted a more stringent ratio of 10:1, requiring 10 participants per item. Given that the questionnaire comprised 20 items, a minimum of 200 responses was deemed sufficient for robust analysis. Furthermore, Comrey and Lee (1992) classify sample sizes as follows: fewer than 100 (poor), 200 (medium), 300 (good), 500 (very good), and 1,000 (excellent). Although an initial target of 1,000 participants was set, the final sample size of 651 respondents was considered adequate for statistical analysis.

Particularly, the Hayes PROCESS macro, which has been shown to yield robust results in larger datasets (Chiu and Ho, 2023), was employed for data analysis. Additionally, Medina-Craven et al. (2020) and Khan et al. (2019) affirm its applicability in studies with substantial sample sizes. Moreover, Wiroko (2021) and Kim (2023) highlight the Process macro’s effectiveness in estimating indirect Effects, reinforcing its suitability for large-scale analyses where sample size constraints may impact statistical validity.

### 4.3 Data analysis

Data were analyzed using the IBM SPSS software package. The statistical significance of the mediation effects was evaluated using the PROCESS plug-in developed by Hayes (2013), which relies on an ordinary least squares regression model and the bootstrap method. Before testing the hypotheses, the validity and reliability of the measures were assessed through analyses of internal consistency, convergent validity, and discriminant validity by using IBM AMOS version 24. Hierarchical regression analyses of the direct effect (c’), total effect (c), and bootstrapped bias-corrected 95% confidence intervals of the indirect effect were computed using the IBM SPSS Process macro version 4.2. These analyses were conducted with 5,000 bootstrapped samples, following the method outlined by Preacher and Hayes (2008).

In this study, Harman’s Single Factor Test was utilized to assess common method bias, given that the same data collection method was employed to examine the relationships between variables. According to this approach, common method bias is indicated when the first factor accounts for more than 50% of the total variance in exploratory factor analysis. The analysis, conducted using IBM SPSS package program, revealed that the first factor accounted for approximately 23% of the total variance, meaning that common method bias was not a concern in this study (Harman, 1967).

## 5 Results

### 5.1 Descriptives

The sample consisted predominantly of female participants (86.8%, *n* = 525), with the largest segment being in the 26–30 age group (28.4%, *n* = 185). Also, 59.1% (*n* = 385) of the participants were married, most of the participants had a bachelor’s degree as their educational background (59.6%, *n* = 388), and 31.8% had an average wage between $763 and $998 (per month).

Regarding their professional roles, 35.3% worked in clinical units, 23.5% in other health units such as physical therapy centers or maternity wards, 14.7% in intensive care units, 11.5% in outpatient clinics, 12% in administrative units, and 2.9% in the operating room. Concerning their job titles, the largest group consisted of primary health professionals such as nurses, midwives, and emergency medical technicians, making up 74.2% of the participants. 22.4% of the participants were other health professionals (e.g., physiotherapist, lab technician), 3.2% were administrative staff (e.g., medical secretary, patient admission officer) and 0.2% were clinic staff (e.g., doctor, dentist, and pharmacist). Among health professionals, 54.1% worked rotating shifts, whereas 45.5% worked regular daytime hours. Concerning patient caseloads, 36.9% handled 26 or more patients daily, 30.9% attended to 5–10 patients, 13.5% managed 11–15 patients, 9.8% had 16–20 patients under their care, and 8.8% took care of 21–25 patients.

Finally, we asked healthcare professionals about the challenges they experience in the hospital environment. Among health professionals, 65.6% reported dealing with a significant workload, 36.3% experienced instances of violence from patients and their families, 33.2% faced frequent difficulties taking breaks due to the demands of their jobs, 31.5% encountered workplace bullying, 26.9% associated disruptions in their work-life balance with night shifts, and 24.7% highlighted various communication issues with colleagues.

### 5.2 Measurement model testing

For testing the measurement model, confirmatory factor analysis (CFA) was conducted using the IBM AMOS v24 software. Two items related to surface acting, one item related to deep acting and one item related to organizational impediments were excluded from the model due to their factor loadings being less than 0.50. When these four items were removed from the model, the fit indices were within acceptable ranges (see Table 1).

**Table 1:** Model fit indices.

Chi-Square	GFI	NFI	TLI	CFI	RMSEA
3.992	0.931	0.920	0.926	0.939	0.068

Table 2 shows descriptive statistics, internal consistency, and correlation values between emotional labor dimensions (surface and deep acting), organizational impediments, and job satisfaction. The internal consistency analysis indicates that the Cronbach’s alpha values of the scales fall within acceptable ranges. In the discriminant validity analysis, AVE values and variance shared between the constructs were compared. Table 2 demonstrates that the correlation values for each pair of constructs are lower than the square root of the AVE values, thereby confirming discriminant validity.

**Table 2:** Discriminant validity results.

	Cronbach alpha	AVE	MSV	Mean	SD	1	2	3	4
1. Surface acting	0.815	0.513	0.139	2.47	0.85	0.716			
2. Deep acting	0.807	0.713	0.139	3.08	1.01	0.373**	0.845		
3. Organizational impediments	0.738	0.480	0.096	3.00	0.97	0.309**	0.072	0.693	
4. Job satisfaction	0.898	0.645	0.038	3.09	0.96	−0.085	0.195**	−0.170**	0.803

AVE, average variance extracted; MSV, maximum shared variance. **p < 0.01.

Convergent validity was evaluated through the examination of factor loadings, average variance extracted (AVE), and composite reliability (CR) values (see Table 3). Following the removal of items with factor loadings below 0.50, all remaining items demonstrated factor loadings exceeding 0.60. The composite reliability (CR) values, ranging from 0.71 to 0.90, fell within the acceptable range. Additionally, AVE values of more than 0.50 indicated that the convergent validity of the construct was achieved (Fornell and Larcker, 1981). Thus, it can be concluded that all constructs possess both reliability and convergent validity.

**Table 3:** Convergent validity results.

	Items	Factor loadings	CR	AVE
Surface acting**	5	0.632–0.819	0.801	0.513
Deep acting*	3	0.852–0.906	0.881	0.713
Organizational impediments*	3	0.621–0.856	0.715	0.480
Job satisfaction	5	0.776–0.893	0.900	0.645

*Represent an item was excluded from the analysis due to its low factor loading (<0.50).

CR, composite reliability; AVE, average variance extracted.

### 5.3 Structural model and hypothesis testing

The significance level for all statistical analyses was established at *α* = 0.01 and *α* = 0.05. Accordingly, results with *p*-values less than 0.01 or 0.05 were deemed statistically significant.

To verify the research hypotheses, we conducted structural equation modeling with IBM SPSS Process macro. Based on Hair et al.’s (2010) study, we determined that the hypothesized model demonstrated a good fit with the observed data. Table 1 provides a clear display of the model fit indices. Table 4 demonstrates the standardized regression path estimates. Accordingly, organizational impediments positively affect surface acting (*β* = 0.332 *p* < 0.001), therefore supporting H1. H2, which hypothesized that organizational impediments positively affect deep acting, is also supported (*β* = 0.094 *p* < 0.044). The results also showed that surface acting had a negative impact on job satisfaction (*β* = −0.130 *p* < 0.005), thus supporting H3. H4, which hypothesized the positive effect of deep acting on job satisfaction (*β* = 0.248 *p* < 0.001), was accepted. In addition, organizational impediments had a negative effect on job satisfaction (*β* = −0.144 *p* < 0.004). Thus, H5 accepted.

**Table 4:** Path estimates.

Path		Estimate	SE	*p*-values	Remarks
H1	Organizational impediments → surface acting	0.332	0.122	0.001	Supported
H2	Organizational impediments → deep acting	0.094	0.091	0.044	Supported
H3	Surface acting → job satisfaction	−0.130	0.051	0.005	Supported
H4	Deep acting → job satisfaction	0.248	0.050	0.001	Supported
H5	Organizational impediments → job satisfaction	−0.144	0.114	0.004	Supported

The mediation analysis conducted with the IBM SPSS Process macro is presented in Table 5. Using Model 4 from the Process macro and the bootstrapping procedure outlined by Preacher and Hayes (2008), we tested the mediation effects of organizational impediments and job satisfaction through surface acting and deep acting. The indirect effects are considered significant if the confidence interval does not include zero. The examination of standardized indirect effects along the hypothesized paths revealed that surface acting completely mediated the relationship between organizational impediments and job satisfaction (*β* = −0.0371; *p* < 0.01; CI.95 = −0.064, −0.013). Thus, the organizational impediments perceived by healthcare professionals at the healthcare institutions were negatively associated with their job satisfaction, and this relationship was fully mediated by surface acting. However, the confidence interval comprised zero, indicating that deep acting did not mediate the relationship between organizational impediments and job satisfaction (β = 0.0174; *p* < 0.01; CI.95 = −0.002, 0.040). These findings indicate that H6 is supported, but H7 is not supported.

**Table 5:** Mediation model.

IV	DV	Standard regression	SE	*t-*value	*p-*value	Model *R*^2^
Direct effects						0.0598
OI	SA	0.268	0.0351	7.0931	0.001	
OI	DA	0.074	0.0409	1.8804	0.060	
OI	JS	−0.093	0.0386	−23.783	0.018	
SA	JS	−0.138	0.0450	−3.2729	0.001	
DA	JS	0.236	0.0386	5.7854	0.001	
Indirect effects		Effect	Boot SE	Boot LLCI	Boot ULCI	
OI → SA → JS		−0.0371	0.0127	−0.064	−0,013	
OI → DA → JS		0.0174	0.0107	−0.002	0,040	

OI, organizational impediments; JS, job satisfaction; SA, surface acting; DA, deep acting.

## 6 Discussion

Emotional labor, defined as the effort an individual exerts to modify their emotions and the way they express them during interactions with others, is a key aspect of emotion management. Demonstrating emotional labor to various organizational stakeholders is a complex and demanding task for employees. This challenge is particularly pronounced for healthcare professionals, who operate in more difficult and complex work environments. This study addresses the issue of organizational impediments, a topic that has not been extensively explored in the existing literature. It investigates the impact of these impediments on emotional labor behaviors (surface acting and deep acting), as well as job satisfaction. Additionally, the study explores whether emotional labor mediates the relationship between organizational impediments and job satisfaction.

To provide context and enhance the significance of our findings, it is essential first to assess the working conditions of healthcare professionals. Our research indicated that healthcare professionals receive relatively low wages, with most participants earning between €693 and €906 per month. This aligns with a recent OECD (2021) report, which highlighted that nurse salaries in Turkey are significantly lower than the OECD average, with an average monthly wage of €872. Given Turkey’s high inflation rate —reported at 71.60% by the Turkish Statistical Institute (TÜİK, 2024) and 113% by the Turkish Inflation Research Group (ENAG, 2024)— these wage levels are considered exceedingly insufficient. Our study reveals that, in addition to low wage levels, a significant number of healthcare professionals face challenges such as insufficient break times (33.2%), difficulties with night shifts (26.9%), excessive workloads (65.6%), workplace bullying (31.5%) and violence from patients and their families (36.3%). These adverse workplace conditions disrupt the work-life balance of healthcare professionals and contribute to communication difficulties in their interactions with others.

In addition to the challenging working conditions previously described, healthcare workers also identified significant organizational impediments within their institutions. Among the 651 healthcare professionals surveyed, 60% reported that their organizations were characterized by an inflexible and rigid structure, with issues such as factionalism, internal groupings, and pervasive gossip being prevalent. These organizational dynamics further exacerbated the already challenging work environment. Given the emphasis that service sector managers place on effective communication with customers to improve performance (Morris and Feldman, 1996), it becomes challenging for healthcare professionals to have job satisfaction and regulate their emotions when they face considerable challenges in the work environment and perceive organizational impediments.

Our findings indicate that organizational impediments contribute to an increase in both surface acting and deep acting, thus hypotheses H1 and H2 are supported. The results suggest that organizational impediments compel healthcare professionals to exert greater effort in managing their emotions, necessitating heightened control over their emotional expressions and behaviors. Consequently, these impediments amplify the effort, planning, and control needed for emotion management, leading employees to either display inauthentic emotions, suppress their true emotions, or internalize the emotions mandated by their roles.

According to Koinis et al. (2015), the emotional well-being of healthcare professionals can be adversely impacted by several factors, including the inherently stressful nature of the profession, workplace stress, insufficient organizational support, role ambiguity, interpersonal conflicts, and individual personality traits. Beyond these factors, the responsibility healthcare professionals bear for human life further predisposes them to occupational stress and burnout (Koinis et al., 2015). Research indicates that a supportive work environment can enhance well-being in roles that require close interaction with clients, thereby reducing stress and the demands of emotional labor (Grandey, 2000; Grandey et al., 2004). Moreover, social support from managers, colleagues, and the organization has been shown to mitigate the detrimental effects of stress associated with emotional labor (Abraham, 1998). Studies have also found that a supportive, collegial, and cooperative organizational climate can reduce the suppression of emotions and alleviate negative outcomes such as emotional dissonance (Ryu et al., 2020). Given that emotional labor is associated with negative work attitudes like burnout and exhaustion (Grandey, 2000; Brotheridge and Lee, 2003; Seçer, 2005; Yin et al., 2019; Yao et al., 2015), it is evident that organizational impediments exacerbate the emotional effort required for emotion management and erode employees’ emotional autonomy.

Our research also highlights the impact of emotional labor dimensions on job satisfaction. The findings suggest that surface acting reduces job satisfaction, while deep acting enhances it, thus hypotheses H3 and H4 are supported. Specifically, when healthcare professionals engage in surface acting by forcing themselves to display positive emotions and behaviors toward patients, they experience lower job satisfaction. Conversely, when they engage in deep acting by genuinely altering their emotions to meet the needs of others and internalizing the value of their actions, they derive greater satisfaction from their job. These results align with existing literature, which indicates that suppressing negative emotions increases stress (Dembroski et al., 1985; Gross, 1998) and that emotion suppression reduces job satisfaction (Diefendorff and Gosserand, 2003). For instance, a study on university employees found that emotional labor elevates job stress and diminishes job satisfaction (Pugliesi, 1999). Similarly, Schaubroeck and Jones (2000) discovered that positive emotional displays boost job satisfaction by fostering positive emotional experiences. Xu et al. (2020) further demonstrated that deep acting helps employees build positive psychological resources over time, thereby positively influencing job attitudes such as job satisfaction and organizational commitment, while surface acting shows no significant relationship with job satisfaction.

Our research findings also indicate that organizational impediments significantly diminish job satisfaction, thus hypothesis H5 is supported. Specifically, impediments such as destructive competition, political conflicts, and rigid regulations have been observed to lower employees’ job satisfaction and hinder their ability to have satisfaction in their work. Previous studies have shown that perceived organizational impediments negatively impact job performance and organizational commitment (Siders et al., 2001; Özbağ et al., 2014). These impediments also contribute to a sense of psychological powerlessness among employees, reducing their autonomy and perceived influence over their work and increasing role ambiguity and role conflict (Özbağ et al., 2014). Furthermore, one of the most detrimental effects of organizational impediments is their adverse impact on employee creativity and organizational innovation (Kimberly, 1981; Amabile et al., 1996; Çekmecelioğlu and Pelenk, 2015).

While the body of research exploring the effects of organizational impediments on job satisfaction, job attitudes, and emotional labor is limited, more attention has been given to the influence of organizational support on these outcomes. The concept of organizational support is grounded in Blau’s (1964) Social Exchange Theory and Gouldner’s (1960) Reciprocity Norm. The central premise of Social Exchange Theory is that individuals are likely to exert greater effort and enhance their performance in response to the opportunities, resources, and rewards provided by the organization. The “reciprocity norm,” a fundamental principle of Social Exchange Theory applied to employee behavior, posits that individuals will respond to positive treatment or goodwill with similar positive behaviors (Cropanzano and Mitchell, 2005; Çetin and Şentürk, 2016; Kerse and Karabey, 2017). Consequently, employees who perceive organizational support are expected to experience higher job satisfaction and improved job performance. Research shows a positive relationship between perceived organizational support and job satisfaction across various sectors (Poon et al., 2007; Mabasa and Ngirande, 2015; Gültekin et al., 2023). In the healthcare sector, studies similarly demonstrate that organizational support enhances employees’ sense of belonging, increases job satisfaction, and strengthens organizational commitment (Chang, 2015).

The findings of our research reveal that surface acting fully mediates the relationship between organizational impediments and job satisfaction, thus hypothesis H6 is supported. This outcome underscores that a restrictive work environment characterized by rigid rules and conflicts compels individuals to suppress their emotions more frequently, which in turn leads to a significant decline in job satisfaction. As previously mentioned, research examining the constraining aspects of the work environment, their relationship with emotional labor, and mediating factors remains limited. However, when job satisfaction is understood as an emotional response to both the job and the work environment, it is unsurprising that a restrictive work environment adversely impacts employees’ emotions, thereby reducing job satisfaction. Conversely, it is expected that a supportive work environment would enhance job satisfaction by facilitating more effective emotional regulation among employees. Indeed, existing studies suggest that perceived organizational support can mitigate the negative impacts of emotional labor, thereby increasing job satisfaction (Hur et al., 2015; Duke et al., 2009). Furthermore, research has shown that emotional labor plays a crucial role in the effect of negative organizational conditions on employees’ well-being. For instance, Carlson et al. (2012) found that an abusive management style exacerbates work–family conflict by increasing the prevalence of surface acting.

The final results indicated that deep acting did not mediate the relationship between organizational impediments and job satisfaction, thereby providing no support for hypothesis H7. Although organizational impediments may compel employees to engage in emotional labor through deep behavior, organizational impediments influence on job satisfaction could be attenuated due to the longer-term and more intrinsic nature of deep behavior compared to surface behavior. Employees who display deep behavior are likely to possess greater psychological resilience, as they are able to internalize the emotions and actions that the organization expects, which may restrict the impact of organizational impediments on their job satisfaction. Furthermore, factors such as high workload and stressful working conditions, particularly in the healthcare sector, may influence how individuals exhibiting deep behavior manage organizational impediments, thus limiting the mediating effect of deep behavior on the relationship between organizational impediments and job satisfaction.

### 6.1 Implications for theory

The Job Demands-Resources (JD-R) Theory provides a framework for understanding the impact of job demands and job resources on employees’ motivation and burnout levels (Demerouti et al., 2001). This study extends the JD-R framework by demonstrating that organizational impediments (e.g., rigid hierarchy, factionalism, conflict) function as job demands for healthcare professionals. These impediments necessitate greater emotional effort, compelling employees to engage in both surface and deep acting. While the JD-R model traditionally posits that job demands contribute to burnout, whereas job resources enhance motivation, the findings of this study refine this perspective by highlighting the distinct effects of different emotional labor strategies on job satisfaction. Specifically, surface acting is negatively associated with job satisfaction, whereas deep acting enhances it. These findings suggest that emotional labor should not be treated as a uniform job demand but rather as a multidimensional construct with divergent consequences for employee well-being.

Similarly, this study expands upon Conservation of Resources (COR) Theory, which postulates that individuals strive to protect and accumulate their resources, while resource depletion induces stress (Hobfoll, 1989). Within this framework, the results indicate that organizational impediments deplete healthcare professionals’ cognitive and emotional resources, increasing the likelihood of surface acting and ultimately leading to a resource loss spiral. As employees exert more energy and effort to navigate these impediments, their job satisfaction declines and their risk of burnout escalates. Furthermore, this study advances the COR theory by contextualizing deep acting within the resource gain principle. Specifically, healthcare professionals may enhance their job satisfaction by imbuing their work with personal meaning or adopting an empathetic perspective, despite the presence of organizational constraints. This suggests that deep acting can serve as a mechanism for positive resource generation, counterbalancing the negative effects of resource depletion.

Moreover, the study provides a novel theoretical contribution by identifying the mediating role of surface acting in the relationship between organizational impediments and job satisfaction. When faced with workplace constraints, employees may resort to surface acting as a short-term strategy to mitigate resource loss. However, in the long run, this coping mechanism may undermine job satisfaction, further reinforcing the detrimental cycle of resource depletion. These insights not only refine the COR framework but also underscore the importance of differentiating between emotional labor strategies in organizational research, particularly within high-stress professional settings such as healthcare.

### 6.2 Implications for practice

Organizational impediments, encompassing structural, managerial, and communication-related factors, can significantly impede healthcare professionals’ ability to work efficiently and derive satisfaction from their roles. To mitigate the adverse effects of these impediments, healthcare institutions should prioritize structural reforms that promote inclusivity and employee engagement in decision-making processes. Specifically, rigid hierarchical structures should be relaxed, facilitating a shift toward a more horizontal organizational framework that enhances participatory management. Such an approach fosters a sense of agency among employees, thereby reducing dissatisfaction and improving overall workplace dynamics. To address managerial challenges, it is crucial to adopt a transparent and equitable management approach that mitigates bias and factionalism. Ensuring fairness in institutional policies and developing mechanisms that uphold equal treatment across all employee levels can contribute to a more cohesive work environment. Moreover, promoting a teamwork-oriented culture through interdepartmental collaboration initiatives can further alleviate the detrimental impact of organizational impediments by enhancing cooperation and reducing workplace conflicts. In terms of communication strategies, establishing effective conflict resolution mechanisms is essential. This can be achieved by providing conflict management training to managers and offering employees access to professional support services, such as Employee Assistance Programs (EAPs). Additionally, fostering transparent communication practices—including the implementation of an open-door policy—can help prevent misinformation and speculation, thereby minimizing the spread of gossip and reinforcing trust between management and staff.

Given the emotional demands inherent in healthcare professions, institutional leaders should take proactive measures to make emotional labor more manageable. One key strategy involves strengthening psychological resilience among healthcare professionals by establishing vocational support groups, mentoring programs, and supervision mechanisms. Providing access to psychological counseling services can also serve as a valuable resource in helping employees cope with emotional stressors. Furthermore, training programs on emotional labor management should be integrated into professional development curricula to raise awareness and enhance healthcare professionals’ ability to navigate emotional demands effectively. Such programs should emphasize empathy development and effective communication skills, enabling employees to engage in emotional labor in a more sustainable and adaptive manner. Workload management is another critical factor in mitigating the negative consequences of emotional labor. To prevent excessive emotional strain, work schedules should be optimized, overtime minimized, and break periods adequately extended when necessary. Additionally, job rotation strategies can be implemented to alleviate the psychological burden associated with repetitive exposure to stressful tasks. Where feasible, flexible working arrangements should be explored to promote work-life balance and reduce emotional exhaustion.

Recognizing and valuing employees’ emotional efforts is also integral to ensuring that emotional labor does not remain invisible. Developing reward and recognition programs that acknowledge emotional contributions—whether through formal performance incentives or informal appreciation mechanisms—can foster a culture of respect and motivation.

Finally, healthcare administrators should cultivate a psychologically safe work environment where employees feel comfortable expressing ideas, making mistakes without fear of punitive consequences, and actively engaging in workplace decision-making. Participatory management strategies can further strengthen employees’ sense of belonging, thereby improving job satisfaction. Moreover, policies that support work-life balance, such as flexible scheduling and enhanced leave policies, should be implemented to ensure employee well-being. Alongside financial incentives, non-monetary recognition strategies, such as personalized appreciation messages and performance awards, can contribute to increased motivation and engagement. Importantly, supportive leadership that prioritizes trust-building and recognizes employees’ emotional needs can serve as a fundamental driver of enhanced job satisfaction and overall organizational well-being.

### 6.3 Limitations and noted directions

In this study, a cross-sectional research design was employed. While cross-sectional designs are valuable for identifying associations between variables, they inherently limit the ability to establish causal relationships (Spector, 2019). For instance, although the study demonstrates the impact of organizational impediments on emotional labor and job satisfaction, it does not allow for definitive conclusions regarding the long-term effects or the directionality of causation. To gain a more comprehensive understanding of the temporal dynamics between these variables, future research should consider adopting longitudinal research designs, which would facilitate the examination of changes over time and provide stronger evidence for causality.

Additionally, in this study, data were collected at a single point in time, which poses challenges in fully eliminating common method variance inherent in the measurement process. Although Harman’s Single Factor Test was employed to assess and control for common method bias, the lack of temporal separation among the independent, mediating, and dependent variables remains a significant limitation. As Podsakoff et al. (2003) have underscored, collecting data at multiple intervals is essential for mitigating common method bias by reducing the inconsistencies and biases that can occur when all measures are obtained from the same source concurrently. Consequently, future research should incorporate a temporal separation strategy in data collection to enhance the validity of the findings and to facilitate a more accurate interpretation of the causal relationships among variables.

Moreover, the study utilized a convenience sampling method, which, while practical, presents limitations in terms of generalizability due to the non-random nature of participant selection (Etikan et al., 2016). Specifically, the reliance on online surveys may have led to selection bias, as healthcare professionals with limited digital access or lower motivation to complete the survey might have been underrepresented. To enhance sample representativeness, future studies could employ random sampling or stratified sampling methods, ensuring a more diverse and inclusive participant pool.

Another important limitation relates to sample size considerations. Although the final sample of 651 participants is statistically sufficient, large samples can increase the likelihood that even small effect sizes become statistically significant, which may obscure their practical relevance (Cohen, 1992). While the Hayes PROCESS macro, utilized in this study, has demonstrated robust performance in large samples, careful interpretation is required to distinguish between statistical and practical significance. Future research should complement statistical significance testing with effect size reporting to provide a more nuanced understanding of the findings’ real-world implications.

Additionally, the study relied on self-report surveys to measure healthcare professionals’ perceptions of emotional labor, organizational impediments, and job satisfaction. However, self-reported data may be susceptible to common method bias and social desirability bias, as participants may consciously or unconsciously distort their responses due to organizational pressures or concerns about social acceptability (Podsakoff et al., 2003). To mitigate potential measurement errors, future studies should consider integrating multiple data sources, such as managerial assessments, peer evaluations, or patient satisfaction data, to enhance measurement validity and reduce response bias.

Furthermore, mediation effects were tested using the Hayes PROCESS macro, which is a widely used tool for estimating direct and indirect effects (Hayes, 2018). While PROCESS provides a powerful approach for mediation analysis, its assumptions regarding linear relationships must be carefully considered. For instance, the relationship between emotional labor and job satisfaction may not necessarily be linear, and potential interaction effects may have been omitted. To address these limitations, confirmatory analyses using alternative modeling techniques, such as structural equation modeling (SEM) or multilevel modeling, could be conducted in future research. These methodological refinements would enhance the validity and reliability of findings and contribute to a more robust theoretical and practical understanding of organizational psychology in the healthcare sector.

### 6.4 Future research

Future research should explore additional mediating and moderating variables to further investigate the relationships among organizational impediments, emotional labor, and job satisfaction within the theoretical frameworks of Job Demands-Resources (JD-R) theory and Conservation of Resources (COR) theory. The JD-R model posits that job demands influence work outcomes through burnout. In this regard, burnout may serve as a mediator in the relationship between organizational impediments and job satisfaction, as increased organizational constraints can contribute to emotional exhaustion among healthcare professionals, subsequently leading to lower job satisfaction. Additionally, future studies could examine whether psychological safety mitigates the adverse effects of organizational impediments. Psychological safety fosters a supportive work environment, allowing employees to navigate emotional labor demands more effectively. Given its potential buffering role, psychological safety could be investigated as a moderating variable that alleviates the negative impact of organizational impediments on surface and deep acting. Furthermore, as deep acting has been found to be positively associated with job satisfaction, work engagement may be another crucial variable to consider in this relationship, particularly within the healthcare sector. Investigating the extent to which work engagement enhances the beneficial effects of emotional labor would provide further insights into mechanisms that support healthcare professionals’ well-being.

Moreover, organizational support may act as a moderator, mitigating the detrimental effects of emotional labor on job satisfaction. COR theory (Hobfoll, 1989) posits that social support serves as a resource that helps employees cope with job-related stressors and reduces resource depletion. Thus, future studies could explore organizational support as a protective factor, potentially weakening the relationship between organizational impediments and surface acting. Another relevant avenue for research involves the role of work–family conflict in the relationship between organizational impediments and burnout. Jobs requiring high emotional labor can exacerbate work–family conflict, further diminishing job satisfaction. Investigating whether work–family conflict functions as a moderator in this dynamic could provide deeper insights into the broader implications of organizational impediments on employees’ personal and professional well-being.

Examining the mediating and moderating mechanisms discussed above would contribute to a more comprehensive understanding of the emotional labor processes among healthcare professionals and develop organizational policies aimed at enhancing workplace well-being and efficiency.

### 6.5 Conclusion

Healthcare workers must possess emotional resilience to deliver high-quality healthcare services and provide emotional support to patients. However, this becomes challenging for healthcare professionals who perceive significant organizational impediments. This study offers significant academic and practical contributions by examining the impact of organizational impediments on the emotional labor processes and job satisfaction of healthcare professionals. The findings indicate that organizational impediments positively influence both surface and deep acting, as they compel employees to exert greater effort, planning, and control in managing their emotions. However, these impediments also exert a negative impact on job satisfaction. Specifically, while surface acting is negatively associated with job satisfaction, deep acting has a positive effect, highlighting the differential effects of distinct emotional labor strategies on employees’ well-being. Furthermore, the study identifies surface acting as a mediating mechanism in the relationship between organizational impediments and job satisfaction, suggesting that compelling healthcare professionals to engage in surface acting may be a primary factor contributing to reduced job satisfaction.

Although prior research has extensively examined the relationship between emotional labor and job satisfaction, the role of organizational impediments in shaping surface and deep acting behaviors has received relatively limited attention. This study addresses this gap by demonstrating how organizational constraints influence employees’ emotional labor strategies and by uncovering the underlying mechanisms through which organizational impediments diminish job satisfaction. In particular, the identification of surface acting as a mediating variable offers a novel perspective on how organizational challenges negatively affect job satisfaction through employees’ emotional labor processes. From the perspective of the Job Demands-Resources (JD-R) Theory, the findings suggest that organizational impediments function as job demands, depleting employees’ emotional resources. Simultaneously, within the framework of Conservation of Resources (COR) Theory, the results illustrate that surface acting contributes to resource loss, thereby lowering job satisfaction, while deep acting serves as a potential resource-gaining process, enhancing job satisfaction. By integrating these theoretical perspectives, this study extends the JD-R and COR theories within the healthcare context, offering sector-specific insights into how organizational impediments influence emotional labor and job satisfaction. These contributions provide a theoretical foundation for future research while also offering practical implications for organizational policies aimed at improving the work environment and well-being of healthcare professionals.

## Data Availability

The raw data supporting the conclusions of this article will be made available by the authors, without undue reservation.
